# Supplementary material for: Triazole-Functionalized Mesoporous Materials Based on Poly(styrene-block-lactic acid): A Morphology Study of Thin Films
Source: Polymers (Basel). 2022 May 31;14(11):2231. doi: 10.3390/polym14112231 (PMC9182962; doi:10.3390/polym14112231)
Supplement: Supplementary file 1 [file polymers-14-02231-s001.zip › polymers-1727336-supplementary.pdf]

Supporting Information

Triazole-Functionalized Mesoporous Materials Based on Poly(Styrene-*Block*-Lactic Acid): A Morphology Study of Thin Films.

Melisa Trejo Maldonado[1,2], Aisha Womiloju[2], Steffi Stumpf[2,3], Stephanie Hoeppener[2,3], Ulrich S. Schubert[2,3], Luis E. Elizalde Herrera[1]*, Carlos Guerrero Sánchez[2,3]*

[1]Centro de Investigación en Química Aplicada, Blvd. Enrique Reyna, No.140, 25100 Saltillo, Coahuila, México.

[2]Laboratory of Organic and Macromolecular Chemistry (IOMC), Friedrich Schiller University Jena, Humboldtstr. 10, 07743 Jena, Germany.

[3]Jena Center for Soft Matter (JCSM), Friedrich Schiller University Jena, Philosophenweg 7, 07743 Jena, Germany.

Content

1.1 2D DOSY NMR spectra of copolymer PS_{113}-*b*-PLA_{118}. 3

1.2 Solvent selection for the copolymer casting 3

1.3 Atomic Force Microscopy (AFM) of untreated and annealed substrates. ... 4

1.4 Solvent vapor annealing (SVA) on silylated substrates. 4

1.5 Synthesis of acetylene-terminated poly (lactic acid) (PLA-Ac) 5

1.6 PLA Hydrolysis of the bulk films 6

1.7 Isolated PS-*b*-PLA based monolith (method 2) 7

1.8 Styrene polymerization by Activators Regenerated by Electron Transfer – Atomic Transfer Radical Polymerization (ARGET-ATRP)................................... 7

1.9 Synthesis of azide-terminated polystyrene (PS-N_3)...................................... 8

1.10 Differential Scanning Calorimetry (DSC) and Thermogravimetric Analysis (TGA) of the synthesized copolymers.. 9

1.1 2D DOSY NMR spectra of copolymer PS_{113}-*b*-PLA_{118}.

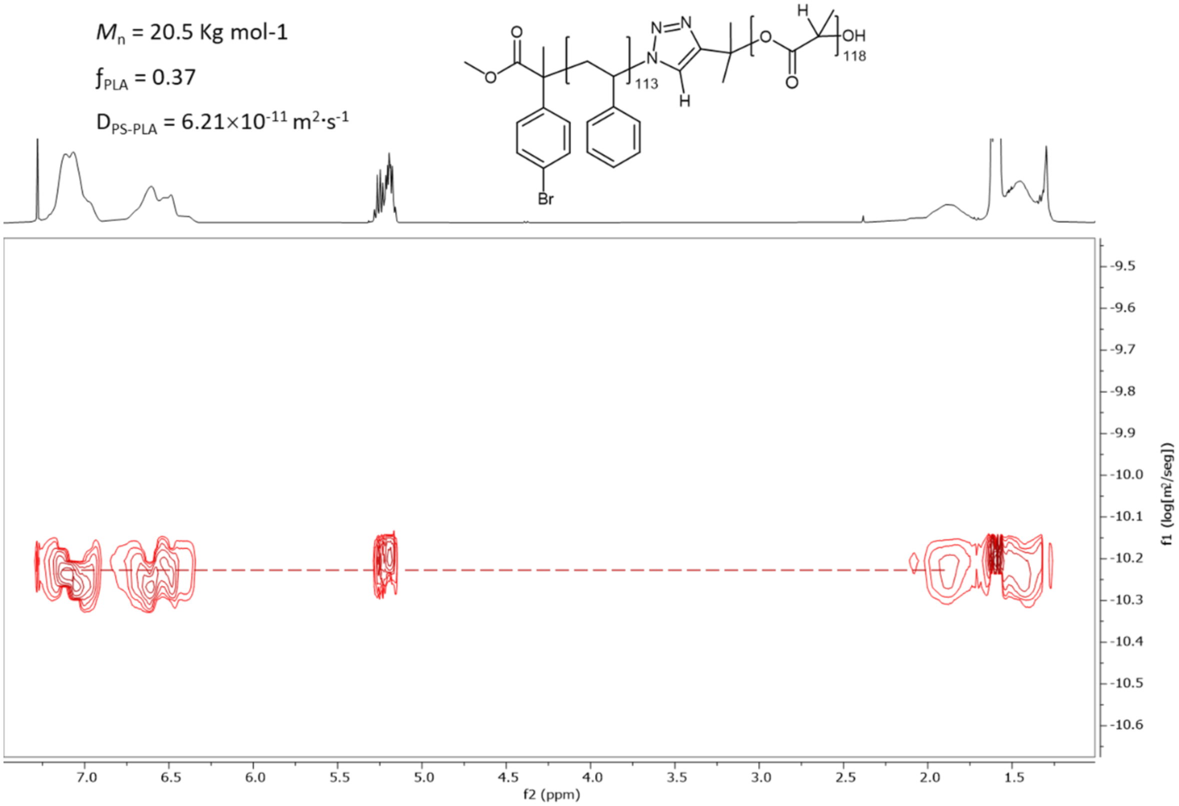

Figure S1. *2D DOSY NMR spectra* of copolymer PS_{113}-*b*-PLA_{118} (400 MHz, r.t., $CDCl_3$).

1.2 Solvent selection for the copolymer casting

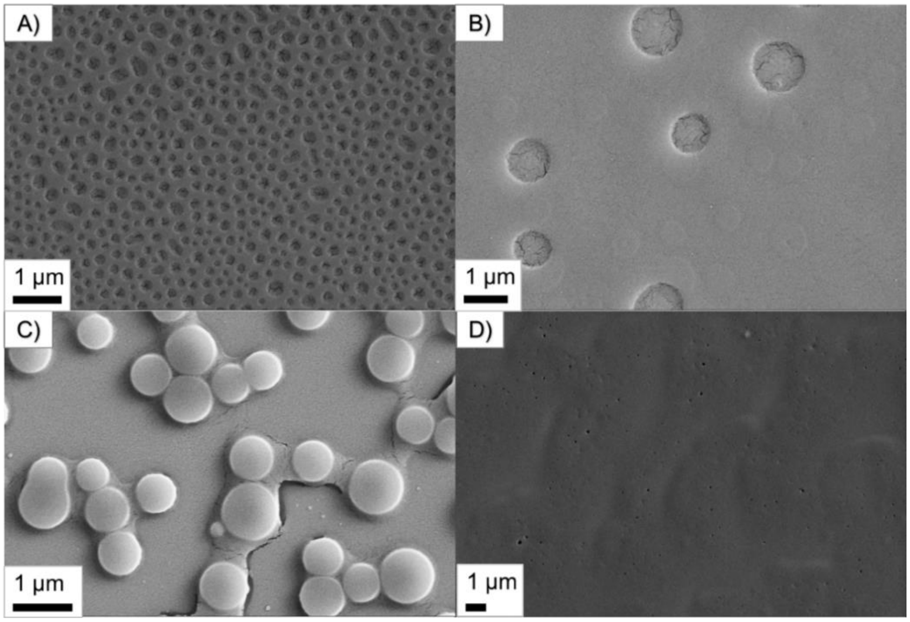

Figure S2. *As-spun silicon wafers with a 2.0 wt.% solution of 1A with different solvents: A) THF, B) CH_2Cl_2, C) dioxane and D) toluene.*

1.3 Atomic Force Microscopy (AFM) of untreated and annealed substrates.

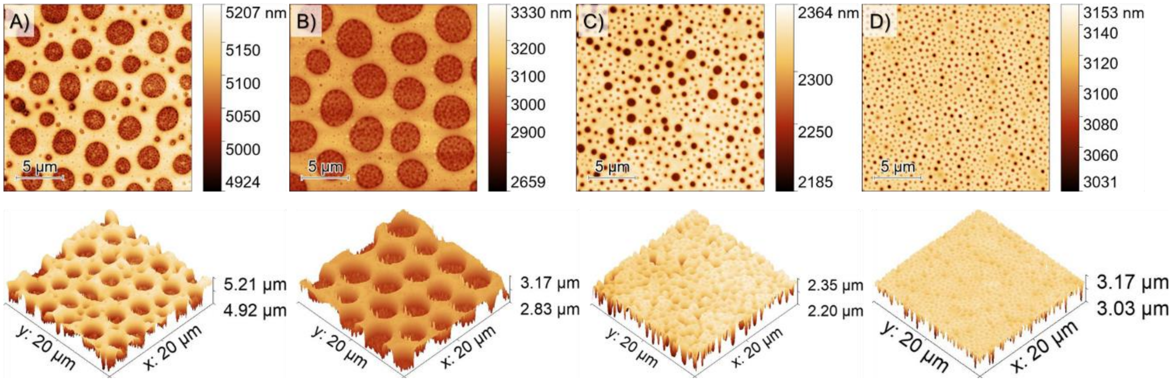

Figure S3. *AFM of the untreated substrates at A) t = 0, B) 15 min, C) 30 min and D) 45 min of annealing with SVA.*

1.4 Solvent vapor annealing (SVA) on silylated substrates.

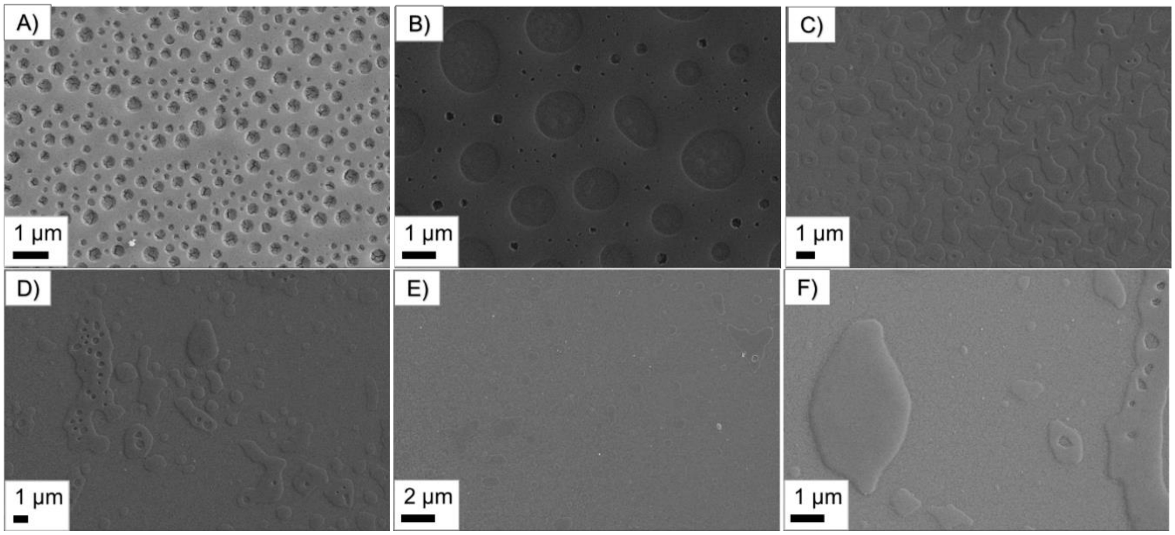

Figure S4. *SVA of treated substrates at longer annealing times A) as-spun, B) 45 min, C) 2 h, D) 4 h, E) 8 h and F) 16 h.*

1.5 Synthesis of acetylene-terminated poly (lactic acid) (PLA-Ac)

1.0 g (6.95 mmol) of 3,6-dimethyl-1,4-dioxan-2,5-dione (D,L.lactide) and 10.1 mg (6.95 mmol) of 4-ethynyl benzoic acid were weighed in a 0.5 – 2.0 mL *Biotage* vial. A minimum amount of toluene was added to the vial and was later freeze-dried overnight to remove water moieties. In a separate vial, 28.1 mg (6.95 mmol) of tin (II) 2-ethylhexanoate were dissolved in 2.0 mL of anhydrous toluene. The solution was degassed by displacing the air with nitrogen stream for 20 min. After degassing, solution was added to the first vial and the mixture was sealed under nitrogen. Reaction was let to occur in a *Biotage* Microwave reactor by placing the vial under the following parameters: *t* = 30 – 90 min, T = 120 °C, 600 rpm and '*Normal*' absorption set-up. The reaction was quenched by placing the open vial inside an ice bath. Product was re-dissolved in a minimum amount of CH_2Cl_2 and precipitated in an excess of cold methanol. A second precipitation was performed using a mixture of hexane/ethanol, 8:2. A white powder was isolated after vacuum drying at 40 °C overnight.

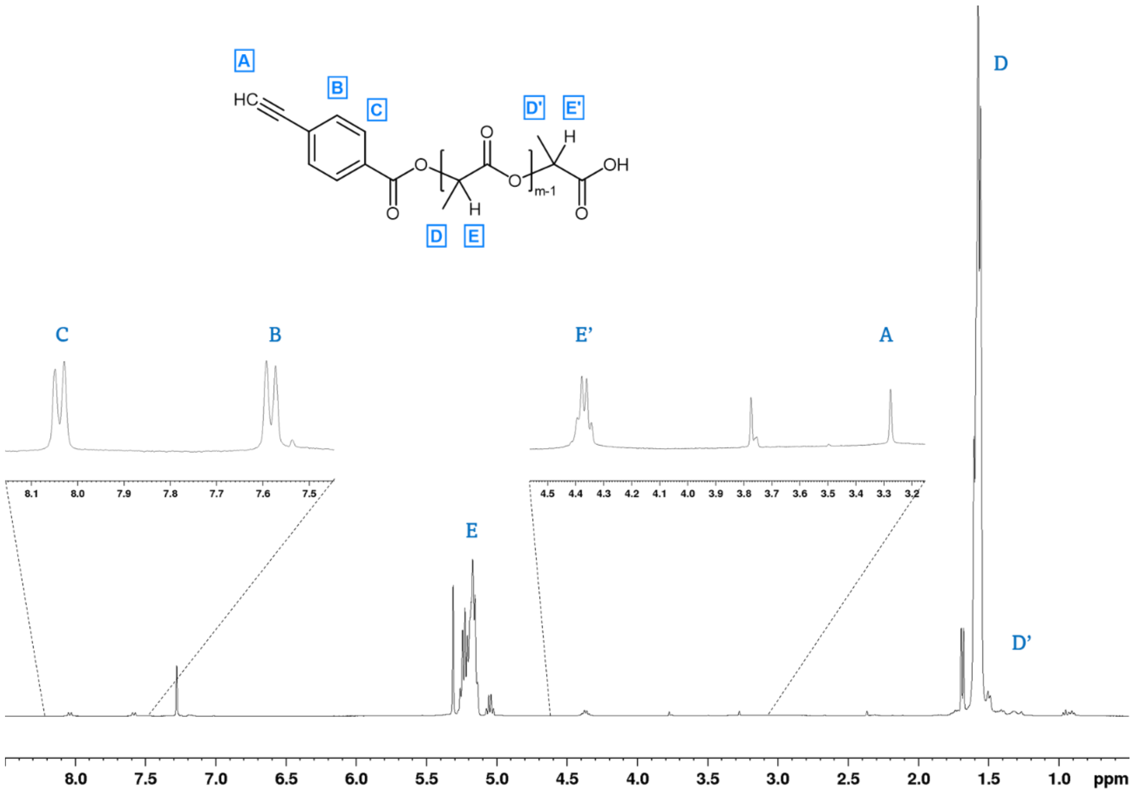

Figure S5. *^{1}H NMR of acetylene terminated polylactic acid (PLA-Ac).*

1.6 PLA Hydrolysis of the bulk films

A fraction of the prepared film was carefully cut into small pieces (~ 2 – 4 mm), several of these pieces were needed to take samples during the hydrolysis. Then, these pieces were immersed in a large volume of an alkaline solution: NaOH 0.5 M (3:2, H_2O: CH_3OH). The mixture was placed inside an oil bath at 60 °C. The hydrolysis was let to occur for several days, and samples were taken at different times. To monitor the hydrolysis, a sample was taken out of the media and extensively rinsed with water and methanol. After briefly drying in the oven the sample was analyzed with ^{1}H NMR and the change in the integral value of PLA repetitive unit was observed (Fig. S5). The final product was freeze-dried prior to the SEM characterization.

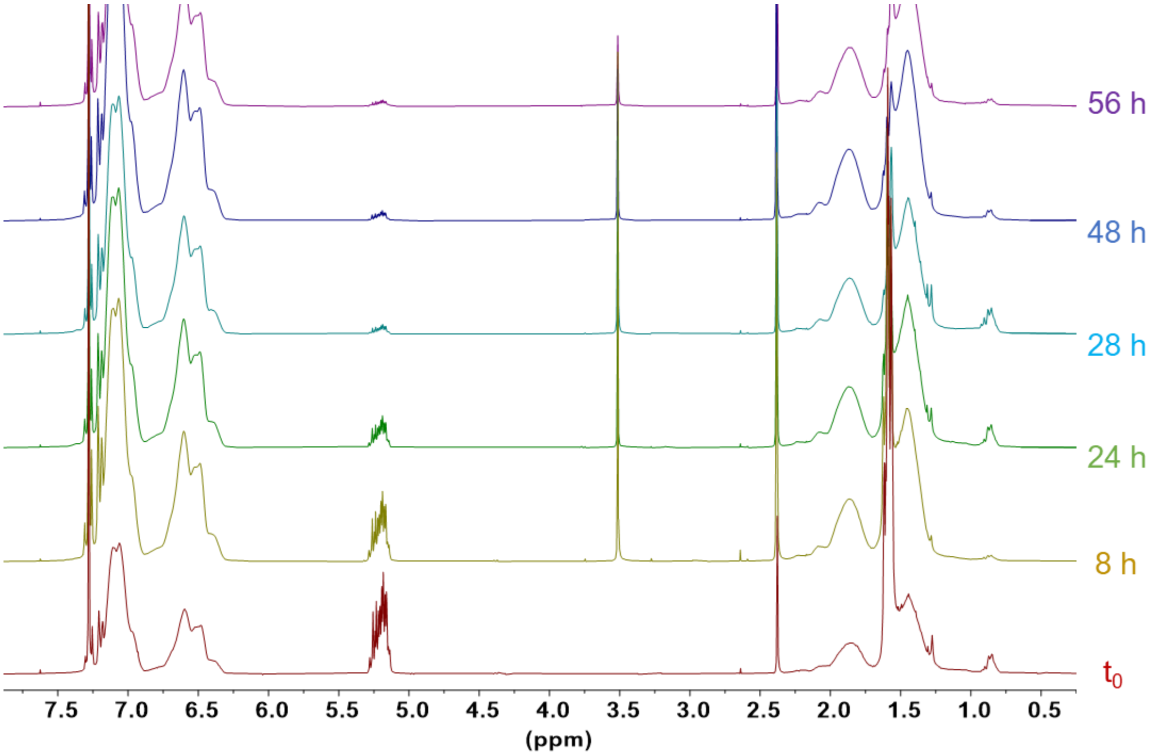

Figure S6. *^{1}H NMR spectra of the hydrolyzed samples at different times (**2A**, method 1).*

1.7 Isolated PS-*b*-PLA based monolith (method 2)

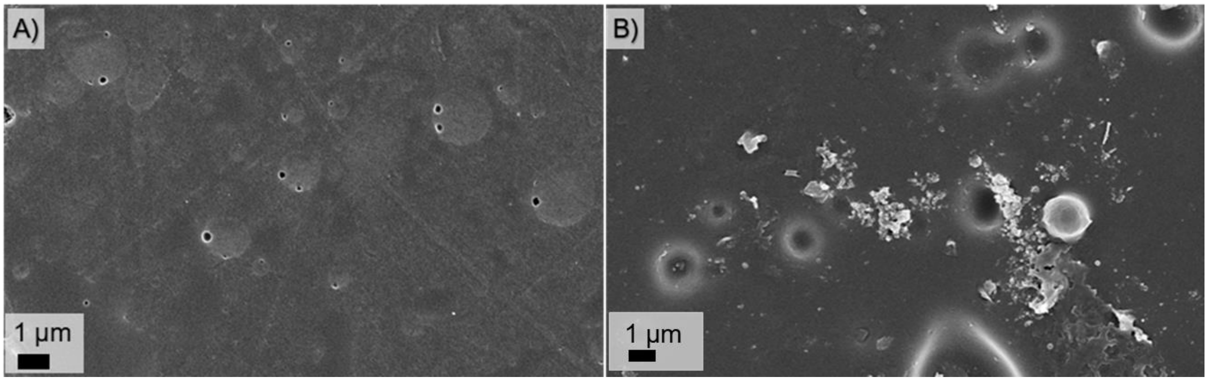

Figure S7. *SEM images of bulk sample (**2B**) A) annealed sample, and B) hydrolyzed sample.*

1.8 Styrene polymerization by Activators Regenerated by Electron Transfer – Atomic Transfer Radical Polymerization (ARGET-ATRP).

14.0 mg (0.06 mmol) of copper bromide (II) ($CuBr_2$), were weighed in a 50 mL Schlenk tube and were promptly placed under a N_2 stream to evacuate the oxygen in the atmosphere. 166.0 µL (0.62 mmol) of tris[2-(dimethylamino) ethyl] amine (Me_6TREN), were dissolved in 2.0 mL of toluene, and added with a degassed micropipette to the Schlenk. This mixture was let to stir to promote the formation of the Cu/Ligand complex. After 10 – 15 min, 10.2 g (89.4 mmol) of styrene and 105.0 mg (0.31 mmol) of methyl 2-bromo-2-(4-bromophenyl) propanoate (MBBP), were diluted in 4.0 mL of toluene and added with a degassed syringe to the Schlenk. Then, 50.3 mg (0.31 mmol) of tin (II) 2-ethynilhexanoate ($Sn(EH)_2$), were diluted in 2.0 mL of toluene and added to the mixture. The resulting solution was degassed after three cycles of freeze-pump-thaw and placed into an oil bath at 100 °C for 24 – 72 h. After completion, reaction was quenched by placing the Schlenk into an ice bath and opened to air. The copper catalyst was removed by eluting the mixture with CH_2Cl_2 through an alumina column. The concentrate was precipitated by triplicate in an excess of methanol A white/yellow solid was isolated after vacuum drying at 40 °C for 16 h.

1.9 Synthesis of azide-terminated polystyrene (PS-N_3)

4.5 g (0.18 mmol) of bromine-end polystyrene (PS-Br), were dissolved in 7.0 mL of *N*,*N* – dimethylformamide (DMF) by stirring constantly. Afterwards, 0.587 g (9.04 mmol) of sodium azide (NaN_3) were added to the solution. The resulting mixture was stirred for 4 days at room temperature. After completion, mixture was precipitated in an excess of distilled water. The solid particles were vacuum filtrated and thoroughly rinsed with water to eliminate the NaN_3 moieties. White pellets were isolated after freeze-drying for 16 h.

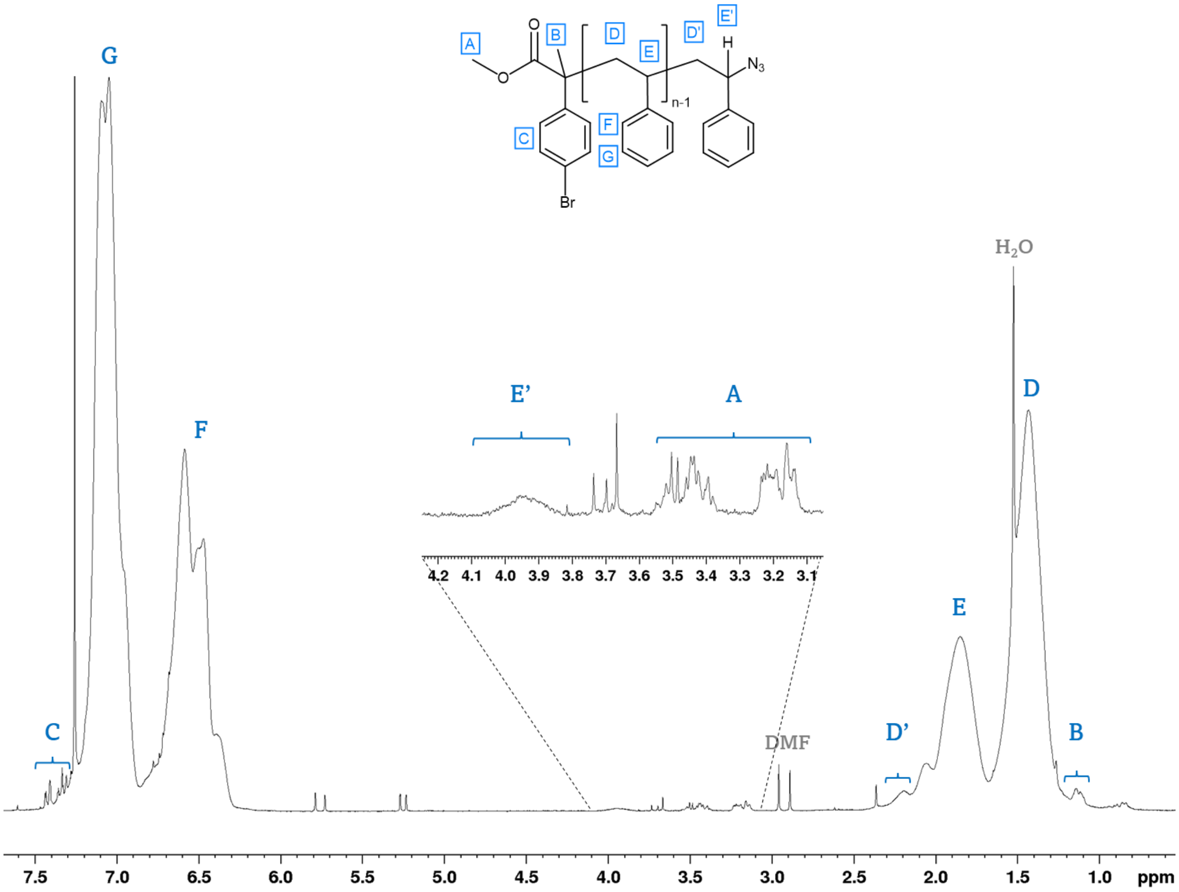

Figure S8. *^{1}H NMR of azide terminated polystyrene (PS-N_3).*

1.10 Differential Scanning Calorimetry (DSC) and Thermogravimetric Analysis (TGA) of the synthesized copolymers.

Table S1. *Thermal features of the synthesized PS_n-b-PLA_m copolymers*

ID	$T_{g\,1\,PLA}$ [a]	$T_{g\,2\,PS}$ [a]	$T_{D\,PLA\,1}$ [b]	$T_{D\,PS\,2}$ [b]
**1A**	48.4	95.4	217.5	385.1
**2A**	54.9	101.8	291.3	391.4
**1B**	51.4	98.7	223.6	378.5
**2B**	51.9	102.5	222.7	387.6
**3B**	50.0	100.7	252.4	389.8

[a] T_g (°C) = average glass transition temperature measured by DSC (-50 – 150 °C, 10.0 K min^{-1}, N_2 20 mL min^{-1}).

[b] T_D (°C) = onset decomposition transition measured by TGA (25 – 600 °C, 10.0 K min^{-1}, N_2 atm).

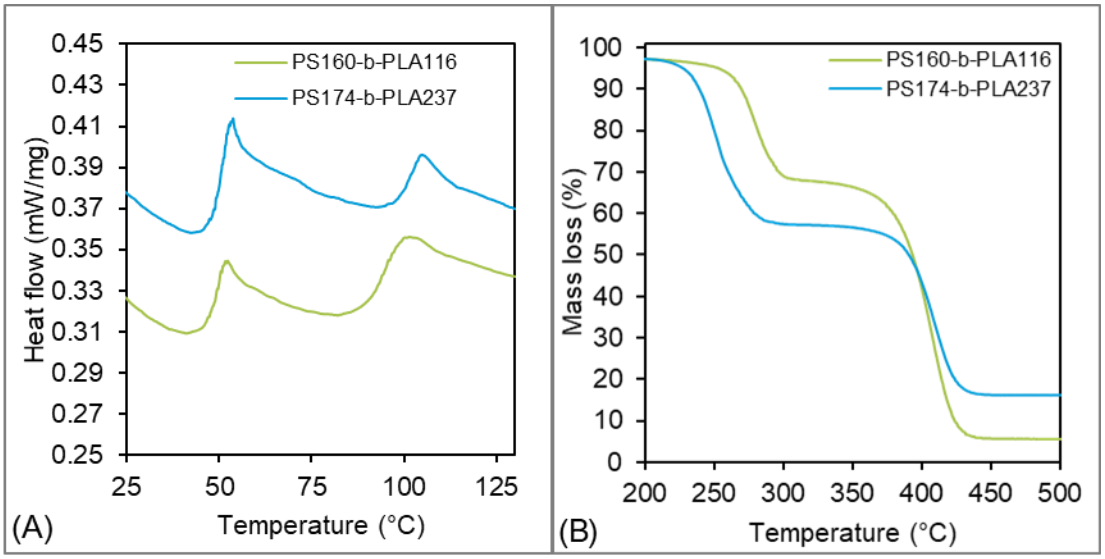

Figure S9. *DSC A) and TGA B) thermograms of copolymers 1A (green line) and 3B (blue line).*
